# *In vitro* assessment of time-dependent changes in red cell cytoplasmic antioxidants of donkey blood preserved in citrate phosphate dextrose adenine 1 anticoagulant

**DOI:** 10.14202/vetworld.2020.726-730

**Published:** 2020-04-19

**Authors:** Okereke Henry Nnamdi, Udegbunam Rita Ijeoma, Nwobi Lotanna Gilbert, Ezeobialu Henry Toochukwu, Udegbunam Sunday Ositadinma

**Affiliations:** 1 Department of Veterinary Surgery, Faculty of Veterinary Medicine, University of Nigeria, Enugu State, Nigeria; 2 Department of Veterinary Physiology and Pharmacology, Faculty of Veterinary Medicine, University of Nigeria, Enugu State, Nigeria; 3 Veterinary Teaching Hospital, Faculty of Veterinary Medicine, University of Nigeria, Enugu State, Nigeria

**Keywords:** blood, citrate phosphate dextrose adenine 1, donkey, malondialdehyde, superoxide dismutase

## Abstract

**Background and Aim:**

Stored blood is continuously exposed to oxidative stress, which affects its antioxidant protective system. Erythrocytes are naturally armed with antioxidant protective capacity. Blood antioxidant system functions to protect the blood cells against oxidative damage by free radicals. However, during storage, blood is continuously exposed to oxidative stress, which affects its antioxidant system. The aim of this work was to investigate alteration in malondialdehyde (MDA) levels, reduced glutathione (glutathione reductase [GSH-Rd]), catalase (CAT), and superoxide dismutase (SOD) activities in stored donkey blood.

**Materials and Methods:**

Blood (250 ml) was drawn from four clinically healthy donkeys into citrate phosphate dextrose adenine 1 blood bags and preserved at 4°C. MDA, GSH-Rd, CAT, and SOD activities were assayed by colorimetric methods, over a period of 42 days.

**Results:**

The result showed that SOD enzyme activities significantly (p<0.05) increased by day 7 post-storage (PS) while MDA levels significantly (p<0.05) increased by day 21 PS. However, activities of GSH-Rd and CAT enzymes decreased (p<0.05) by day 21 PS. Pearson’s product-moment correlation showed a negative correlation between the levels of MDA and enzymatic antioxidant markers (CAT and GSH-Rd).

**Conclusion:**

The findings revealed that GSH-Rd and CAT are the primary antioxidant defense markers in donkey red blood cells. The observed alterations in these principal antioxidants suggest a 14days optimum keeping time of donkey blood for blood banking purposes.

## Introduction

Storage media available for the preservation of blood meant for transfusion purposes maintain the viability and oxygen-carrying capacity of the collected blood [1-4]. At present, anticoagulants recommended for use include citrate phosphate dextrose (CPD), CPD adenine (CPDA-1), acid citrate dextrose, storage media for blood, heparin, and a whole lot of others [1,5]. The Food and Drug Administration permits blood to be stored up to 42days before transfusion with the use of CPDA-1 anticoagulant. Citrate in the media functions to bind the ionized calcium present in whole blood and thus inhibits several steps in the coagulation cascade while dextrose allows generation of adenosine triphosphate (ATP) through glycolytic activity. Orlov and Kakouti [2] posited that the maintenance of ATP levels correlates with red cell viability. The addition of adenine to the preservative CPDA-1 promotes red cell ATP production, which, in turn, lengthens the shelf-life of stored blood. More so, the presence of phosphorus in the preservative enables the blood to maintain a normal oxygen dissociation curve [1,6].

Blood recipients usually have the need to increase their oxygen-carrying capacity, and in such conditions, blood collected in blood bags are either transfused immediately (fresh blood) or preserved over a period of time (stored blood) [4,6]. Stored blood cells undergo biochemical, molecular, metabolic, and biomechanical alterations which occur within the erythrocytes, leading to energetic compromise, which is characterized by ATP and 2,3-diphosphoglycerate (2,3-DPG) depletion as well as loss of red cell membrane integrity [7]. The red blood cell (RBC) membrane damage is caused by the free radicals mediated by lipid and protein oxidation [2,8].

The defense mechanism of blood cells against oxidative damage is their cell membrane antioxidant system consisting of superoxide dismutase (SOD), reduced glutathione (glutathione reductase [GSH-Rd]), and catalase (CAT). Under normal physiological conditions, there is a balance between RBC antioxidant system and free radicals [2,8-10]. Blood storage lesion occurs in conditions when the blood has been in the storage medium for a long time. In such conditions, blood antioxidant system may not be able to protect the blood cells against oxidative damage by free radicals and this has a negative effect on blood quality during storage [11]. In our locality, due to the increasing need for transfusion therapy in animals, including donkeys and the unavailability of specially designed blood bags for these breed of animals, the common practice is to make use of commercially available human blood bag with CPDA-1 preservatives. However, to date, no empirical study has evaluated the changes in the principal cellular RBC membrane antioxidants of donkey blood preserved with commercially available blood bags meant for humans.

## Materials and Methods

### Ethical approval

The experimental protocols used in this study were approved by the Ethics Committee of the University of Nigeria, Nsukka, and conform with the Guide for the Care and Use of Agricultural Animals in Research and Teaching of University of Nigeria, Nsukka, Enugu State, Nigeria.

### Study period and Animals

The duration of this study was from May to July 2019. Four adult male donkeys used for this study were judged to be healthy on the basis of clinical and hematological examinations. They were managed semi-intensively at the Veterinary farm of the Faculty of Veterinary Medicine, University of Nigeria, Nsukka, Enugu State, Nigeria.

### Methodology

Two hundred and fifty milliliters of blood each were drawn from four clinically healthy donkeys into humans (infant) CPDA-1 blood bags (Richter^®^, China). The samples were aseptically collected within 20min, thoroughly but gently mixed with the anticoagulant; immediately, the blood was drawn from the jugular vein of the donkeys and also after filling the bags. Blood was preserved at 4°C and parameters assayed on days 0, 7, 14, 21, 28, 35, and 42, and levels of MDA and activities of GSH-Rd, CAT, and SOD were measured by spectrophotometry.

### Preparation of hemolysate

A volume of 1ml of the blood sample was centrifuged at 2000×g for 20min and the plasma and buffy coat discarded. The RBC was washed 3times in cold phosphate-buffered saline (pH7.45). A1:10 hemolysate dilution in ice-cold distilled water was used for the determination of CAT activity. A1:20 hemolysate in ice-cold distilled water was used for MDA (thiobarbituric acid reactive substance [TBARS]) and SOD measurement. A1:20 hemolysate in 2.7 mM EDTA and 0.7 mM 2-mercaptoethanol was used for the determination of GSH-Rd levels. The hemoglobin (Hb) concentrations of the blood and hemolysate were determined based on the cyanmethemoglobin method using a spectrophotometer (CHEM-5V3; Erba, Mannheim, Germany). The concentration of Hb (g/dl) was determined by comparing against standard solution of cyanmethemoglobin.

### Erythrocyte CAT test

Erythrocyte CAT activity was determined according to the method described previously by Sinha [12]. In this method, dichromate in acetic acid is reduced to chromic acetate when heated in the presence of hydrogen peroxide (H_2_O_2_), with the formation of perchromic acid as an unstable intermediate. Hydrogen peroxide concentration is directly proportional to the concentration of chromic acetate that is produced from the reaction. The chromic acetate produced is measured spectrophotometrically at 570nm. Briefly, 0.04ml of the hemolysate was added to 2.96ml of H_2_O_2_ (0.2 M)-phosphate buffer (0.01 M, pH7). From this mixture, 2ml of dichromate/acetic acid reagent was used to stop the reaction with an interval of 1min. The tubes were heated at 100°C for 10min, cooled, and then centrifuged at 2500×g for 5min to remove precipitated proteins, and the changes in absorbance were recorded at 570nm against the reagent blank using a spectrophotometer (Jenway 6305; Jenway, Essex, UK). Astandard curve was prepared with 0-200 mMol H_2_O_2_. Erythrocyte CAT activity was expressed in mMol H_2_O_2_ decomposed/min/g Hb.

### Erythrocyte reduced glutathione (GSH-Rd) test

Reduced glutathione in the erythrocytes was determined according to the method described previously [13]. The principle is based on the formation of a yellow-colored complex when dithionitrobenzene (DTNB) reacts with the acid-soluble sulfhydryl groups (non-protein thiols) of which more than 93% is reduced glutathione. The absorbance of the colored complex was measured at 412nm. Avolume of 0.5ml of the hemolysate was mixed with 0.1ml of 25% TCA and kept on ice for a few minutes. This was then centrifuged at 3000g for 10min and 0.3ml of the supernatant was mixed with 0.7ml of 0.2 M sodium phosphate buffer (pH8) and 2ml of 0.6 mM DTNB (Sigma, St. Louis, MO, USA). After 10min, the yellow color obtained was measured at 412nm using a spectrophotometer (Jenway 6305; Jenway, Essex, UK) against a reagent blank. Astandard graph was prepared using different concentrations (0-100 µMol) of GSH (Sigma, St. Louis, MO, USA). The GSH content was calculated with the help of this standard graph and expressed as µMol/g Hb.

### Erythrocyte malondialdehyde (MDA) test

Erythrocyte MDA level, an end product of lipid peroxidation, was measured based on the method of Stocks and Dormandy [14]. Thiobarbituric acid reacts with MDA to produce a stable chromogen that is quantified by spectrophotometry. The color intensity of the chromogen is measured at 532nm, which is directly proportional to MDA content. Lipid peroxidation in erythrocytes was quantified by measuring the formation of TBARS based on the method of Stocks and Dormandy (1971). Equal volume of hemolysate (0.5ml) was mixed with 20% trichloroacetic acid (1:1) and incubated at room temperature. Samples were centrifuged at 2500×g for 10min. Then, 1.0ml of 1% thiobarbituric acid was added to the supernatant and samples were placed in a boiling water bath (100°C) for 15min. The contents were cooled on ice and centrifuged for 15min at 2500×g. The absorbance (A) of the supernatant was read at 532nm against a reagent blank using a spectrophotometer (Jenway 6305; Jenway, Essex, UK). Astandard graph was prepared using different concentrations (0-20 nMoles) of MDA (Sigma, St. Louis, MO, USA). Lipid peroxidation (TBARS) level in the erythrocytes was expressed in nMol/g Hb.

### Erythrocyte SOD test

SOD activity in the erythrocytes was determined according to the method developed by Misra and Fridovich [15]. The test is based on the ability of SOD to inhibit the autoxidation of epinephrine to adrenochrome at pH10.2. Avolume of 0.5ml of hemolysate was diluted with an equal volume of distilled water, followed by the addition of 0.25ml of ice-cold ethanol and 0.15ml of ice-cold chloroform. This was thoroughly mixed using a cyclomixer and then centrifuged at 2500×g for 10min. The supernatant was mixed with 1.5ml of carbonate buffer (0.05 M, pH10.2) and 0.5ml of 0.5 mM EDTA solution. The reaction was initiated by the addition of 0.4ml of 3 mM epinephrine (Sigma, St. Louis, MO, USA) and the change in absorbance per minute was measured at 480nm against a reagent blank. The enzyme unit was defined as the change in absorbance per minute at 50% inhibition of epinephrine to adrenochrome by SOD. An enzyme calibration curve was prepared using 0-195 units of SOD (Sigma, St. Louis, MO, USA). SOD activity was expressed in U/g Hb.

### Statistical analysis

The calculated values were entered into Microsoft Excel sheet for documentation and analysis. SPSS version21 (IBM Corp., NY, USA) was used for statistical analysis. Mean, standard error of mean, and confidence intervals were determined for each analyzed variable. Data collected were statistically compared within group using one-way analysis of variance. The least significant difference *post hoc* test was used to separate the variant means at p<0.05 as statistically significant. Pearson’s product-moment correlation was run to determine the magnitude and direction between the oxidative and antioxidative enzymes changes that occurred during storage. Correlation of variant means at p<0.01 and p<0.05 was used as statistically significant.

## Results

This study showed that there was a progressive significant (p<0.05) decrease from the baseline in GSH-Rd and CAT enzyme activity from day 21 to day 42 of blood storage. Furthermore, SOD enzyme activity significantly (p<0.05) decreased by day 7 blood storage (Table-1), but no significant (p>0.05) variation was observed from SOD results between days 7 and 28 of blood storage. The findings of MDA levels revealed a progressive significant (p<0.05) increase throughout the blood storage duration (Table-1).

**Table-1:** Changes in the mean oxidative and antioxidative parameters of CPDA-1 stored canine blood (Mean±SE).

Days	MDA (nMol/gHb)	SOD (U/gHb)	GSH-Rd (μMol/gHb)	CAT (μMol/min/gHb)
0	20.9±0.7^a^	970.3±61.9^a^	6.21±0.2^a^	5.9±1.0^a^
7	31.7±4.3^b^	1093.2±56.3^b^	6.32±0.3^a^	6.0±0.1^a^
14	45.3±1.9^c^	992.0±123.1^b^	6.66±0.3^a^	6.0±0.0^a^
21	72.1±3.6^d^	1059.8±123.8^b^	2.30±0.1^b^	3.1±0.1^b^
28	73.1±1.1^e^	1020.3±39.6^b^	4.95±0.1^b^	3.2±0.9^b^
35	71.9±1.1^e^	746.0±49.8^c^	3.85±0.0^c^	3.1±0.1^b^
42	72.0±3.6^e^	611.1±49.4^c^	4.08±0.1^c^	3.1±0.4^b^

Different superscripts

a,b,c,d in a column indicate significant difference between the mean at the level of probability: (p<0.05). MDA=Malondialdehyde, GSH-Rd=Glutathione reductase, CT=Catalase, SOD=Superoxide dismutase, SE=Standard error

Pearson’s product-moment correlation coefficient was used to determine the magnitude and direction of association between the oxidative marker (levels of MDA) and the antioxidant enzymes (SOD, CAT, and GSH-Rd). The result of the test showed a negative correlation between the MDA levels and activities of CAT (r=−0.717, n=28, p<0.000) and GSH-Rd (r=−0.608, n=28, p<0.001). However, no significant variation was recorded between the levels of MDA and activities of SOD (r=−0.363, n=28, p<0.06).

## Discussion

Blood stored in standard blood banking conditions for transfusion or experimental purposes undergo time-dependent changes [3]. These changes include loss of membrane and cytosolic antioxidant enzymes, alterations in lipids and structural membrane proteins, loss of 2,3-DPG, and ATP [2]. These changes possibly lead to unsatisfactory post-transfusion reactions in the patient [4].

In this study, levels of MDA, as well as activities of CAT, GSH-Rd, and SOD, were assayed on days 0, 7, 14, 21, 28, 35, and 42. The major enzymatic systems against free radicals and peroxides include activities of SOD, CAT, and GSH-Rd [16,17]. Blood antioxidant defense mechanisms maintain the free radical levels at physiological limits by acting as oxidation inhibitors. However, during blood storage, oxidative changes remain one of the important findings. Studies in dog and human blood preserved in standard blood banking conditions revealed time-dependent changes vis-a-vis loss of GSH, CAT, and SOD [4,8]. Hanachi and Shemshaki [18] reported that this could be due to decreased efficacy of the antioxidant defense mechanism, leading to increased reactive oxygen production from free radicals.

SOD converts superoxide anion to hydrogen peroxide, which is subsequently transformed to water by GSH-Rd or CAT. In blood, these antioxidant defense mechanisms offer protection to membrane lipids and proteins against oxidative damage caused by free radicals, free hemin, and iron [19]. It had earlier been documented that in stored blood, SOD, CAT, and GSH-Rd protect the blood cells and Hb majorly from oxidative changes [19]. In this study, activities of GSH-Rd and CAT were significantly decreased by the 21^st^day of storage compared to the baseline values, whereas the MDA levels in the stored blood significantly increased from day 7 to day 42. Findings from enzymatic activities of SOD revealed a significant increase by day 7 from the baseline reading. The recorded concurrent decrease in reduced glutathione activity alongside concurrent with an increase in oxidative modification of membrane lipids and proteins (MDA), may destabilize the membrane skeleton, thereby compromising RBCs survival.

The result of this study is dissimilar to the findings of Marjani *et al*. [17], Deyhim *et al*. [8] on human blood, and that of Udegbunam *et al*. [4], while comparing the oxidative changes seen in human and canine blood stored with commercially available human blood bag. In these earlier studies, decreased activities in CAT and glutathione were recorded after 7-14days of storage while this study revealed time-dependent change from day 21 of blood storage. Nonetheless, the findings of Aslan *et al*. [20] on the levels of oxidative marker (MDA) showed a significant increase from day 7 till the end of the study, which agreed with this finding. Furthermore, Udegbunam *et al*. [4], Gultekin *et al*. [16], Marjani *et al*. [17], and also reported decreased SOD activity at days 7-14 of storage, whereas a contradictory finding of significantly increased SOD activity from day 7 to day 28 was recorded in this study. Red cells are normally protected against oxidative injury by the reduced rate of spontaneous oxidation of hemoglobin. The NADH-dependent cytochrome-b5 reductase reduces methemoglobin back into oxyhemoglobin, while cytosolic antioxidants (SOD, GSH, and CAT) neutralize the generated reactive oxygen species [2,19]. During blood storage, there are increased acidosis and oxygen partial pressure due to the increased spontaneous oxidation of hemoglobin to methemoglobin, and as a result, all the outlined protective mechanisms are impaired. In the face of all these activities, there is reduced antioxidant defense exposing the blood cells to oxidative stress, which is the predominant cause of blood storage lesions [2].

Pearson’s product-moment correlation of the oxidative marker (MDA) with the antioxidant activity (SOD, GSH-Rd, and CAT) to determine the primary antioxidant defense in stored blood showed an association between MDA levels and activities of CAT and GSH-Rd but no association between the activities of SOD and levels of MDA. From a previously reported work on human blood, glutathione [8] and CAT [19] were documented as the primary antioxidants. The findings of this work agree with the findings of Constantinescu *et al*. [19], in which CAT and reduced glutathione jointly protected Hb from oxidative damage. Glutathione catalyzes the reduction of hydrogen peroxide by reduced glutathione (GSH-Rd) and protects Hb from oxidative breakdown. The erythrocyte antioxidant defense potential decreases in correlation with the increase in oxidative stress.

## Conclusion

This study established that during blood storage, time-dependent changes in red cytosolic antioxidant activities occurred. This study also showed that the primary antioxidant defense of donkey blood during storage was GSH-Rd and CAT.

## Authors’ Contributions

OHN and URI conceived the idea. OHN and EHT wrote the first draft of the manuscript. OHN, URI, USO, and NLG were involved in the design and carried out the experimental work. OHN, URI, and EHT were involved in the analysis. The manuscript was critically read, revised, and approved for submission by all the authors.
